# Nurse-led shared decision-making for hemodialysis vascular access: a randomized controlled trial

**DOI:** 10.3389/fmed.2026.1878576

**Published:** 2026-07-03

**Authors:** Fan Yang, Wenhua Yang, Fangfang Lu, Tao Liang, Yanzhi Zhang

**Affiliations:** 1 Department of Hemodialysis, Linquan County People’s Hospital, Fuyang, China; 2 Department of Emergency Center, Linquan County People’s Hospital, Fuyang, China

**Keywords:** Decisional Conflict Scale, hemodialysis, nurse guidance, shared decision-making, vascular access

## Abstract

**Objective:**

To evaluate the efficacy of a nurse-led stepwise shared decision-making (SDM) model, guided by the Decisional Conflict Scale (DCS), for vascular access management in hemodialysis patients.

**Methods:**

In this randomized controlled trial, 120 hemodialysis patients requiring vascular access establishment or reconstruction were randomly allocated (1:1) via block randomization to an experimental or control group (*n* = 60 each). Control patients received standard health education. The experimental group received a nurse-led SDM intervention integrating DCS assessment and tailored decision aids. The primary outcome was the change in decisional conflict, measured by the DCS score. Secondary outcomes comprised preoperative state anxiety, decision satisfaction, arteriovenous fistula (AVF) selection rates, and 6-month postoperative complications. Post-intervention continuous outcomes were analyzed using Analysis of Covariance (ANCOVA) to adjust for baseline covariates.

**Results:**

Post-intervention, the experimental group demonstrated significantly lower decisional conflict (adjusted mean difference [AMD]: −18.74, 95% CI [−25.58, −11.91], *p* < 0.001, partial eta-squared = 0.20) and preoperative anxiety (AMD: −5.23, 95% CI [−6.31, −4.16], *p* < 0.001, partial eta-squared = 0.44) than the control group. Furthermore, the experimental cohort exhibited higher decision satisfaction (AMD: 3.69, 95% CI [2.32, 5.06], *p* < 0.001, partial eta-squared = 0.19), a higher AVF selection rate (80.0% vs. 58.3%, *p* = 0.022), and significantly lower rates of 6-month postoperative complications.

**Conclusion:**

The nurse-led shared decision-making model significantly reduces decisional conflict and state anxiety while promoting AVF selection when adjusting for baseline covariates. By utilizing a structured, stepwise approach, this model supports objective, patient-centered vascular access decisions in the hemodialysis population.

## 1 Introduction

Vascular access is the “lifeline” for maintenance hemodialysis (HD) patients, as it directly determines the effectiveness of hemodialysis treatment, the quality of life of patients, and long-term prognosis (1). With the rising global incidence of end-stage renal disease (ESRD), the number of ESRD patients requiring dialysis is projected to double to 5.4 million by 2030 (2), which makes the demand for safe and effective vascular access increasingly urgent. Clinically, the main types of vascular access for HD patients include autologous arteriovenous fistula (AVF), arteriovenous graft (AVG), and central venous catheter (CVC) (3). Among them, AVF is recognized as the preferred long-term vascular access for HD patients by guidelines worldwide (4), due to its advantages of long service life, low complication rate, and low cost. The establishment and maintenance of vascular access involve a complex interplay between professional medical parameters and individual patient preferences. The primary influencing factors determining vascular access viability are physiological and anatomical, such as vessel caliber and cardiovascular status, which are assessed exclusively by physicians to establish clinical safety (5). However, secondary influencing factors—including fear of puncture pain, body image concerns, and lifestyle demands—heavily dictate final patient acceptance and adherence (6). In the traditional medical model, doctors often adopt a paternalistic decision-making approach focused primarily on anatomical feasibility, where patients passively accept the recommended treatment plan without fully resolving their secondary psychosocial concerns. This one-way decision-making model easily leads to poor postoperative compliance and insufficient psychological expectations of complications, ultimately affecting the doctor-patient relationship.

Recently, both in China and internationally, nurse-led advanced chronic kidney disease (CKD) and vascular access clinics have successfully incorporated general AVF education into their management protocols, consequently leading to a gradual increase in AVF creation rates during the peri-dialysis period (6). Despite these improvements, a critical gap remains: standard educational pathways typically fail to accurately identify the specific psychological key points driving a patient’s decisional conflict. Routine clinical practices lack a structured mechanism to differentiate whether a patient’s hesitation stems from an information deficit or an unresolved conflict in personal values. Therefore, a standardized and operable implementation path is urgently needed to quantitatively assess this decisional conflict and target nursing interventions accordingly.

Shared decision-making (SDM) is a patient-centered medical decision-making model that emphasizes the full exchange of information between doctors, nurses and patients, and joint participation in the decision-making process based on the patient’s values and preferences (7). As the medical staff who have the most frequent contact with HD patients, nurses have a natural advantage in building a bridge between doctors and patients, exploring patients’ hidden needs and values, and providing continuous health guidance (8). In recent years, SDM has been gradually applied in the field of chronic disease management, but its application in vascular access selection for HD patients is still relatively limited, and specific operational protocols remain scarce (5, 9). Currently, most SDM interventions rely solely on the broad application of educational decision aids (10), which lacks the diagnostic precision needed to resolve these underlying psychological barriers (11), resulting in insufficient pertinence of the intervention measures.

To solve this problem, the primary novelty of this study lies in innovatively introducing the internationally recognized Decisional Conflict Scale (DCS) (12) not merely as a postoperative evaluation metric, but as an upfront diagnostic tool to tailor the SDM process. Alongside a newly developed “vascular access pros and cons comparison table,” a nurse-led stepwise guided SDM model was constructed. This included four key links: baseline assessment of decisional conflict, objective information provision, value clarification, and multi-disciplinary decision confirmation. The purpose of this study is to provide a structured, evidence-based clinical framework for the management of vascular access in HD patients, alleviate patients’ decisional conflict, improve decision quality and satisfaction, and ultimately optimize the prognosis of patients.

## 2 Methods

### 2.1 Sample size, study objects, and ethics

This study was designed as a single-center, parallel-group randomized controlled trial (RCT). The trial design and reporting rigorously adhered to the Consolidated Standards of Reporting Trials (CONSORT) guidelines. The required sample size was determined prior to data analysis using G Power software (version 3.1). Based on previous literature evaluating shared decision-making interventions in chronic care, we anticipated a medium effect size (Cohen’s d = 0.5) for the primary outcome (Decisional Conflict Scale scores). To achieve 80% statistical power at a two-tailed significance level of *α* = 0.05, a minimum of 51 participants per group was required. Accounting for an anticipated attrition rate of 15% over the 6-month follow-up period, we established a target enrollment of 60 participants per group, yielding a total sample size of 120.

This study was conducted within the hemodialysis center of a tertiary hospital. To ensure high-quality and consistent intervention delivery, the SDM interventions were administered exclusively by four specialized nephrology nurses. Each of these nurses possessed a minimum of 5 years of clinical hemodialysis experience and had achieved specialty certification in vascular access care. A total of 120 HD patients who planned to establish or reconstruct long-term vascular access in our hospital from June 2023 to June 2025 were selected by convenient sampling. The inclusion criteria were meeting the indications for HD and needing to establish or reconstruct long-term vascular access; aged ≥ 18 years old; clear consciousness, with basic reading and communication abilities; and voluntarily participating in this study and signing the informed consent form. Patients requiring the reconstruction of long-term vascular access were explicitly included alongside incident patients because prior access failures frequently exacerbate psychological distress and decisional uncertainty. The necessity to choose a new access modality after a traumatic failure makes the structured value clarification provided by this SDM model highly relevant and clinically necessary for this specific subgroup.

The exclusion criteria were complicated with severe cardiovascular and cerebrovascular diseases or expected lifespan < 6 months, and with severe mental cognitive disorders (defined objectively as a score of < 24 on the Mini-Mental State Examination [MMSE] (13, 14) administered during baseline screening). This study was reviewed and approved by the Ethics Committee of People’s Hospital of Linquan County (Approval No. SL-YJ2026-28), and all patients provided written informed consent prior to participation. The trial was prospectively registered at the Chinese Clinical Trial Registry^1^ (Registration No. ChiCTR2300070272).

### 2.2 Study tools

Decisional Conflict Scale (DCS): This study adopted the DCS originally developed by O’Connor (15) to measure the decisional pressure and uncertainty patients experience when facing clinical treatment options. The tool includes 16 items divided into 5 core dimensions: uncertainty, feeling informed, values clarity, support, and effective decision-making. Responses are recorded on a 5-point Likert scale, and the raw score is converted using the following formula: standardized score = (sum of all 16 items / 16) × 25. The standardized score ranges from 0 to 100 points. Generally, a score < 25.0 points indicates efficient decision-making; a score > 37.5 points signifies significant decisional conflict or delayed decision-making, with higher scores being positively proportional to the severity of the conflict. In this study, the Cronbach’s *α* coefficient of the scale was greater than 0.90, indicating excellent internal consistency.

To operationalize this generic scale for the specific clinical context of hemodialysis without altering its validated 16-item structure, the DCS was anchored to the specific modalities of vascular access (AVF, AVG, and CVC). During administration, the generic items were explicitly framed around specialty-specific trade-offs. For example, items assessing “Feeling Informed” were operationalized to reflect the patient’s grasp of the 8 core dimensions of access care (e.g., infection risk, lifespan, cannulation experience), while “Values Clarity” items required patients to specifically weigh practical lifestyle trade-offs, such as balancing the aversion to needle punctures against the risk of severe catheter-related infections and bathing restrictions.

Self-Rating Anxiety Scale (SAS): Patient preoperative anxiety levels were evaluated using the SAS developed by Zung (16), which is widely applied in clinical settings to quantify the severity of subjective anxious feelings and somatic symptoms. This instrument comprises 20 items, scored on a 4-point frequency scale (ranging from 1 = “a little of the time” to 4 = “most of the time”), with 5 positively worded items requiring reverse scoring. The standardized index score is calculated by the following formula: standard score = raw total score × 1.25 (retaining the integer part). The clinical interpretation of the standard score is defined as follows: < 50 points indicates normal status (no apparent anxiety), 50–59 points indicate mild anxiety, 60–69 points indicate moderate anxiety, and ≥ 70 points indicate severe anxiety. In this study, the scale demonstrated high reliability, with a Cronbach’s *α* coefficient exceeding 0.85.

Satisfaction with Decision Scale (SWD): To assess patients’ contentment with their final vascular access choice and the shared decision-making process, this study utilized the authoritative SWD scale developed by Holmes-Rovner et al. (17). This concise tool consists of 6 items (e.g., “I am satisfied with my decision”). Responses are measured using a 5-point Likert scoring system ranging from 1 (strongly disagree) to 5 (strongly agree). The total score is calculated by directly summing the scores of the 6 items, yielding a possible range of 6 to 30 points. The score is evaluated as a continuous variable where higher total scores represent a greater level of satisfaction with the healthcare decision. In this study, the Cronbach’s *α* coefficient of the scale was greater than 0.85, confirming good internal consistency.

The Vascular Access Pros and Cons Comparison Table (Supplementary Table S1) referred to the evidence-based guidelines provided by the National Kidney Foundation’s Kidney Disease Outcomes Quality Initiative (KDOQI) (18): Revised jointly by nephrology experts, specialist nurses and patient representatives, it horizontally compares AVF, AVG and CVC in 8 dimensions in the form of pictures and texts, including “Appearance & Mobility,” “Expected Lifespan,” “Time to Readiness,” “Cannulation Experience,” “Infection Risk,” “Thrombosis Risk,” “Daily Maintenance” and “Long-term Impact.” The tool is easy to understand and can help patients quickly grasp the characteristics of different vascular access types.

### 2.3 Randomization, allocation concealment, and blinding

A total of 120 enrolled participants were randomly assigned in a 1:1 ratio to either the experimental group (*n* = 60) or the control group (*n* = 60). To ensure rigorous study design, block randomization was performed by an independent clinical statistician who had no clinical involvement in patient recruitment or care. The randomization sequence was generated via computer software (SPSS version 26.0) with a fixed block size of 4, without stratification for demographic characteristics or baseline access types.

To guarantee strict allocation concealment, the generated assignments were sealed within sequentially numbered, opaque, sealed envelopes (SNOSE). An independent research assistant, blinded to the sequence generation process, was responsible for screening and enrolling participants. This research assistant opened the envelopes sequentially to reveal the group assignment only after a participant had fully provided written informed consent and completed all baseline clinical assessments.

Due to the nature of the nurse-led educational intervention, it was not possible to blind the participating patients or the nurses delivering the intervention. However, to minimize detection bias, the outcome assessors who collected the follow-up data and the statistician conducting the final data analysis were strictly blinded to group allocation.

### 2.4 Intervention methods

The experimental group implemented the nurse-led stepwise guided SDM model based on DCS. The intervention was led by senior responsible nurses who had received special training on SDM. To ensure standardized execution, all participating nurses completed a comprehensive 10-h training curriculum prior to patient enrollment. This curriculum covered SDM theoretical frameworks, practical application of decision aids, and role-playing exercises focused on non-directive communication. The SDM intervention was delivered as a single, dedicated face-to-face session lasting approximately 30 to 45 min. Recognizing the importance of a patient’s support network in chronic care decisions, family members or primary caregivers were strongly encouraged to participate in this session. To maintain intervention fidelity and standardize the process across different nurses, interactions were guided by a standardized conversational script—particularly utilizing standardized prompts during the value clarification step. Furthermore, 10% of all SDM sessions were randomly audited by the head nurse using a fidelity checklist to ensure strict adherence to the protocol. The intervention was specifically divided into 4 steps:

(1) Prerequisite medical screening and baseline assessment. Prior to the SDM intervention, all patients underwent a routine physiological evaluation by the attending physician, including vascular ultrasound mapping, to assess anatomical suitability for AVF creation. The medical order issued by the physician defined the specific vascular access options that were clinically viable and safe for each individual patient. The subsequent nurse-led SDM process was strictly limited to navigating choices among these medically cleared options. Following this essential medical gatekeeping, the nurse guided the patient to fill in the baseline DCS scale to identify specific decision-making pain points, and formulated a personalized communication strategy based on the assessment results; (2)Provision of objective information. The nurse provided the patient with the “Vascular Access Pros and Cons Comparison Table” (Supplementary Table S1) and explained the characteristics of the three types of vascular access in detail with neutral and non-inductive language combined with physical models, ensuring that the patient fully understood the advantages and disadvantages of each option; (3)Stepwise guidance and value clarification. The nurse used questions to guide the patient to express their preferences. For example: “Between not needing puncture but not being able to take a bath, and needing to endure puncture pain but having a long service life, which one do you value more?” The nurse recorded the patient’s tendencies in a value list to help them sort out their true needs; (4) Multi-disciplinary team decision confirmation. The nurse presented the patient’s clarified value list and preferred vascular access option to the nephrologist and vascular surgeon. At this stage, the physician’s primary role was to act as the clinical gatekeeper. The doctors cross-referenced the patient’s psychosocial preferences against objective physiological data, specifically the vascular ultrasound mapping results, to rigorously evaluate surgical feasibility. The physician confirmed the medical safety of the patien’s preferred plan, ruling out any absolute physiological contraindications. Once clinical viability was verified by the physician, the final decision was solidified in alignment with both medical principles and the patient’s values. Finally, the patient signed the informed consent form, and targeted follow-up was carried out after the operation.

### 2.5 Observation indicators

(1) Decisional Conflict Scale (DCS): The level of decisional conflict was evaluated as the sole primary endpoint, compared between the two groups before and after the intervention. *Secondary outcomes*: (2) State Anxiety (SAS) score: The level of state anxiety was compared between the two groups before and after the intervention; (3) Decision satisfaction (DS): The validated Satisfaction with Decision (SWD) scale was used to evaluate patients’ contentment with the shared decision-making process and the final vascular access selected; (4) Distribution of vascular access selection: The proportion of the actual type of vascular access implemented in the two groups was recorded; and (5) Incidence of complications: All suspected vascular access-related complications occurring during the 6-month follow-up were independently adjudicated by a specialized vascular surgeon who was strictly blinded to the patients’ randomization group allocation. To ensure methodological rigor, the diagnostic criteria were standardized as follows: Stenosis was formally diagnosed via Doppler ultrasound, defined as a > 50% reduction in the luminal diameter compared to the adjacent normal vessel segment, accompanied by a peak systolic velocity ratio (PSVR) > 2.0; Aneurysmal dilatation was defined as a localized enlargement of the access vein to more than twice the normal diameter of the adjacent venous segment; and Puncture-related circulatory failure was clinically defined as the inability to achieve a prescribed dialysis blood flow rate of > 200 mL/min for two consecutive dialysis sessions specifically due to cannulation difficulties. Other complications, including infection, thrombosis, and bleeding, were diagnosed according to the standard clinical definitions established by the KDOQI Clinical Practice Guidelines for Vascular Access.

### 2.6 Statistical methods

Data analysis was performed using SPSS version 26.0 following the intention-to-treat (ITT) principle. Continuous variables were expressed as mean ± standard deviation, while categorical variables were presented as frequencies and percentages. To rigorously account for any potential baseline differences, Analysis of Covariance (ANCOVA) was utilized to compare postoperative continuous outcomes (including DCS and SAS scores) between the two groups, adjusting for baseline pre-intervention scores as covariates. Prior to conducting ANCOVA, the data were rigorously tested for fundamental statistical assumptions. Residual analysis was performed, and the Shapiro–Wilk test confirmed the normality of the residuals for all continuous outcomes (*p* > 0.05). Levene’s test verified the homogeneity of variances across the intervention and control groups (*p* > 0.05). Furthermore, the assumption of homogeneity of regression slopes was confirmed, indicating no significant interaction between the covariates (baseline scores) and the intervention group (*p* > 0.05). Regarding missing data, all 120 randomized patients completed the 6-month follow-up without any attrition or protocol deviations; therefore, the dataset was complete, and missing data imputation methods were not required.

Furthermore, to robustly quantify the magnitude of the intervention’s effects, Adjusted Mean Differences with 95% Confidence Intervals (CIs) and partial eta-squared values were calculated and reported for all continuous outcomes analyzed via ANCOVA.

To rigorously control the family-wise error rate specifically across the primary outcome evaluations, a Bonferroni multiplicity correction was applied to the Decisional Conflict Scale (DCS) comparisons. Because the DCS analysis involved 6 continuous comparisons (1 total score and 5 sub-dimensions), the threshold for statistical significance for these specific outcomes was adjusted to *p* < 0.0083 (0.05/6). All remaining secondary outcomes (including SAS, SWD, access selection, and complications) were evaluated at the standard nominal significance level of alpha = 0.05. All statistical tests were two-sided.

## 3 Results

### 3.1 Comparison of baseline data between the two groups

A total of 120 patients were included in this study, including 60 cases in the experimental group and 60 cases in the control group. All 120 randomized participants strictly adhered to the study protocol and successfully completed all outcome assessments throughout the 6-month follow-up period. Because there were zero instances of participant dropout, missing data, or protocol deviation, missing data imputation methods were not required. Consequently, the final analysis evaluating the intention-to-treat (ITT) population included the complete, original randomized cohort (*n* = 120) without the need for data estimation.

**Figure 1:**
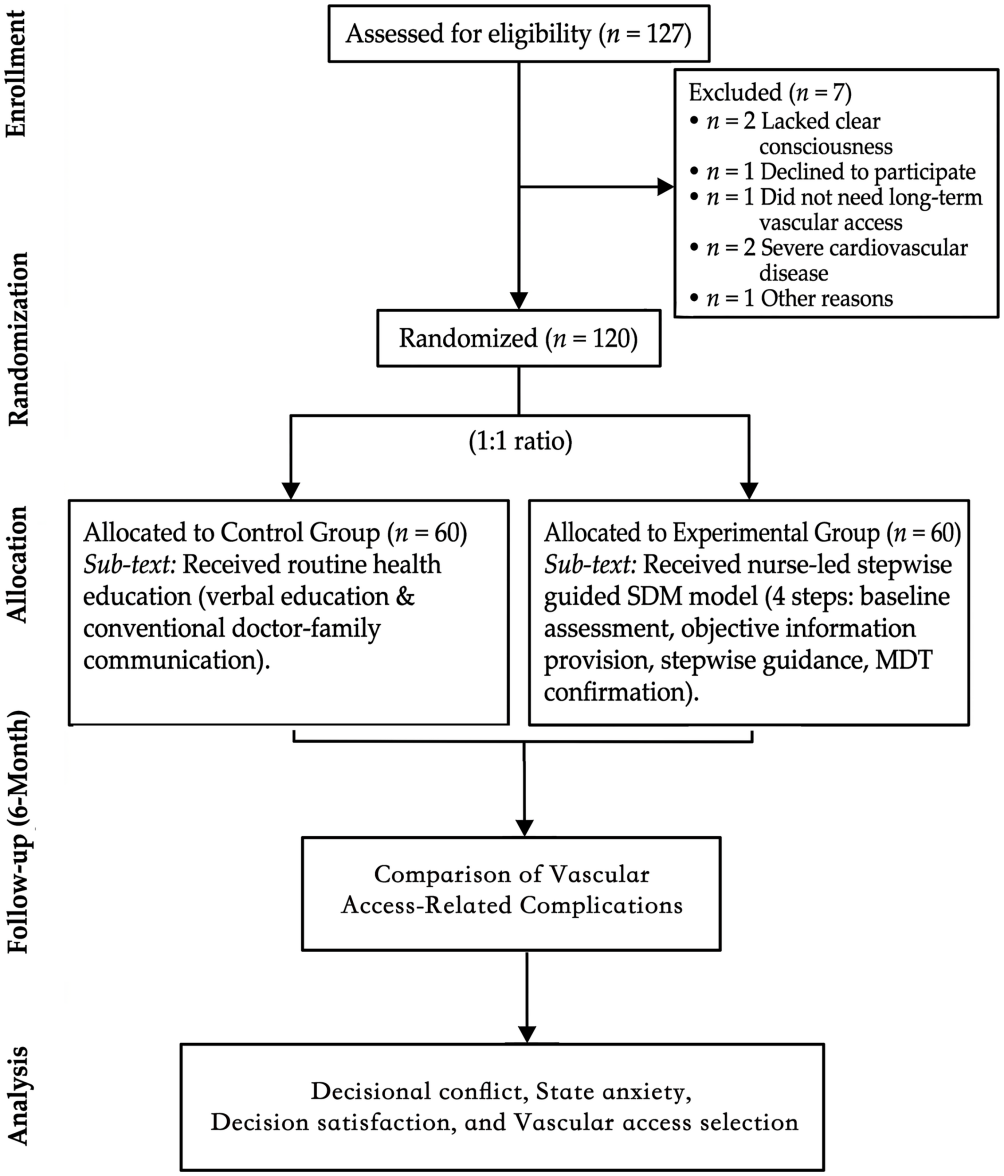
Flow diagram of patient recruitment, randomization, and follow-up. Among the 127 hemodialysis patients assessed for eligibility, 120 were randomized (1:1) into the control group (*n* = 60; routine health education) or the experimental group (*n* = 60; nurse-led stepwise guided shared decision-making model). No patients were lost to follow-up at 6 months. Final analyses were conducted on all 120 participants according to the intention-to-treat principle.

Figure 1 shows the study flow chart. Baseline homogeneity between the two groups was rigorously evaluated across gender, age, dialysis age, comorbidities (diabetes history, hypertension history, cardiovascular complications), laboratory indicators (hemoglobin, serum albumin), and social demographic data (education level, living status, main caregiver, medical payment method). Statistical analysis showed that there were no statistically significant differences in the above baseline indicators between the two groups (all *p* > 0.05), indicating that the two groups of patients had good comparability (Table 1).

**Table 1:** Comparison of baseline characteristics between the two groups (*n* = 120).

Variable	Control group (*n* = 60)	Experimental group (*n* = 60)	Statistic (*t*/*χ*^2^)	*p* value
Gender (Male/Female)	34/26	32/28	0.134	0.714
Age (years)	62.5 ± 8.4	63.1 ± 7.9	−0.403	0.688
Dialysis vintage (months)	24.5 ± 12.3	26.1 ± 13.5	−0.678	0.499
Diabetes history [*n* (%)]	28 (46.7)	30 (50.0)	0.133	0.715
Hypertension history [*n* (%)]	42 (70.0)	45 (75.0)	0.373	0.54
Cardiovascular complications [*n* (%)]	15 (25.0)	18 (30.0)	0.373	0.54
Hemoglobin (g/L)	108.2 ± 12.5	110.1 ± 11.8	−0.856	0.394
Serum albumin (g/L)	36.4 ± 4.2	35.8 ± 3.9	0.811	0.419
Education level (Junior high & below/Senior high & above)	38/22	36/24	0.144	0.707
Living status (Living alone /Not living alone)	12/48	10/50	0.227	0.637
Primary caregiver (Spouse/Children/Others)	35/18 / 7	37/14 / 9	0.816	0.665
Medical payment method (Insurance/Self-pay/Others)	41/12 / 7	32/20 / 8	0.316	0.204

### 3.2 Comparison of decisional conflict (DCS) scores between the two groups before and after intervention

Table 2 presents the continuous outcomes of the Decisional Conflict Scale (DCS) pre- and post-intervention. To rigorously account for baseline variances, Analysis of Covariance (ANCOVA) was employed with the respective pre-intervention scores serving as covariates. The analysis revealed that the experimental group demonstrated a statistically significant reduction in the adjusted post-intervention DCS total score compared to the control group (Adjusted Mean Difference = −18.74, 95% CI [−25.58, −11.91], *F* = 29.48, *p* < 0.001, Eta squared = 0.20). Furthermore, significant improvements were consistently observed across all five DCS sub-dimensions in the experimental cohort, including uncertainty (*F* = 114.27), feeling informed (*F* = 69.49), values clarity (*F* = 318.83), support (*F* = 107.29), and effective decision-making (*F* = 166.72) (all *p* < 0.001). Crucially, these differences remained robust and statistically significant after applying the Bonferroni multiplicity correction (significance threshold strictly adjusted to *p* < 0.0083), confirming a substantial reduction in decisional conflict following the nurse-led SDM intervention (Table 2; Supplementary Tables S2, S3).

**Table 2:** Comparison of decisional conflict scale (DCS) scores and decision-related indicators between the two groups.*

Dimension	Group	Baseline Score (Mean ± SD)	Post-intervention (Mean ± SD)	Adjusted Post-intervention (EMM)	Adj Mean Diff [95% CI]	*F* value	*p* value	Partial Eta-squared
Uncertainty	Control	4.85 ± 2.44	4.55 ± 2.95	4.63	Ref	114.27	< 0.001	0.49
	Experimental	4.65 ± 3.26	2.22 ± 2.14	2.14	−2.49 [−2.95, −2.03]			
Informed	Control	5.68 ± 2.56	4.55 ± 2.95	4.63	Ref	69.49	< 0.001	0.37
	Experimental	5.50 ± 3.37	2.97 ± 2.43	2.89	−1.73 [−2.15, −1.32]			
Values clarity	Control	6.17 ± 2.80	5.50 ± 3.04	5.56	Ref	318.83	< 0.001	0.73
	Experimental	6.03 ± 3.27	2.97 ± 2.43	2.91	−2.65 [−2.94, −2.35]			
Support	Control	5.37 ± 2.48	4.55 ± 2.95	4.54	Ref	107.29	< 0.001	0.48
	Experimental	5.38 ± 3.40	2.22 ± 2.14	2.22	−2.32 [−2.76, −1.88]			
Effective decision	Control	8.37 ± 2.48	7.73 ± 3.20	7.57	Ref	166.72	< 0.001	0.59
	Experimental	8.73 ± 3.60	4.68 ± 2.65	4.84	−2.73 [−3.15, −2.31]			
Total score	Control	47.63 ± 19.77	42.08 ± 23.55	42.14	Ref	29.48	< 0.001	0.2
	Experimental	47.37 ± 26.27	23.45 ± 18.33	23.4	−18.74 [−25.58, −11.91]			

*Adjusted post-intervention scores (Estimated Marginal Means) were calculated using Analysis of Covariance (ANCOVA) with the respective baseline scores entered as covariates. The scores of the 16 items are summed, divided by 16, and then multiplied by 25 to convert the total into a standardized score ranging from 0 to 100. SE, Standard Error; SD, Standard Deviation; EMM, Estimated Marginal Means.

### 3.3 Comparison of self-rating anxiety scale (SAS) and satisfaction with decision scale scores

In addition to decisional conflict, patient anxiety levels were evaluated using the Self-Rating Anxiety Scale (SAS). Following the standard scoring protocol, the reported values reflect the standardized index scores. An Analysis of Covariance (ANCOVA) was subsequently performed to compare the post-intervention SAS standard scores between the two groups, rigorously adjusting for preoperative baseline anxiety levels as a covariate. The results demonstrated a highly significant reduction in state anxiety for patients receiving the nurse-led SDM intervention. The adjusted post-intervention SAS standard score (Estimated Marginal Mean) for the experimental group was 41.40, which was significantly lower than the 46.64 observed in the standard care control group (Adjusted Mean Difference = −5.23, 95% CI [−6.31, −4.16], *F* = 92.27, *p* < 0.001, Eta squared = 0.44). This finding robustly indicates that the stepwise guided SDM model effectively alleviates preoperative anxiety regarding vascular access establishment (Table 3; Supplementary Table S4).

**Table 3:** Comparison of self-rating anxiety scale (SAS) and satisfaction with decision scale scores between the two groups.*

Outcome	Group	Baseline Score (Mean ± SD)	Post-intervention (Mean ± SD)	Adjusted Post-intervention (EMM)	Adj Mean Diff [95% CI]	F value	*p* value	Partial Eta-squared
SAS total score	Control	54.67 ± 23.02	47.52 ± 23.41	46.64	Ref	92.27	< 0.001	0.44
	Experimental	52.88 ± 27.25	40.52 ± 26.55	41.4	−5.23 [−6.31, −4.16]			
Satisfaction (SWD)	Control	22.08 ± 8.32	23.50 ± 7.44	22.74	Ref	28.32	< 0.001	0.19
	Experimental	19.73 ± 9.34	25.67 ± 6.21	26.43	3.69 [2.32, 5.06]			

*Standard score: Calculated by multiplying the raw score by 1.25 and retaining the integer part. SE, Standard Error; SD, Standard Deviation. EMM, Estimated Marginal Means.

Furthermore, patient satisfaction with the decision-making process was assessed using the 6-item Satisfaction with Decision (SWD) scale. Treating the SWD score as a continuous outcome, an ANCOVA was conducted to adjust for baseline satisfaction or expectations regarding the upcoming decision. The results indicated that the experimental group achieved a significantly higher adjusted post-intervention satisfaction score (Estimated Marginal Mean = 26.43) compared to the standard care control group (Estimated Marginal Mean = 22.74). This difference was statistically significant (Adjusted Mean Difference = 3.69, 95% CI [2.32, 5.06], *F* = 28.32, *p* < 0.001, Eta squared = 0.19), underscoring that the shared decision-making model fostered greater patient contentment and alignment with their final vascular access choices (Table 3; Supplementary Table S5).

### 3.4 Distribution of final vascular access selection in the two groups

In terms of the final selection of vascular access, the experimental group had a higher proportion of implementing the “AVF first” principle, with the establishment proportion of autologous arteriovenous fistula (AVF) being 80.0%, which was significantly better than 58.3% in the control group; while the proportion of choosing central venous catheter (CVC) decreased significantly from 21.7% in the control group to 6.7% in the experimental group, and the difference in distribution between groups was statistically significant (*p* = 0.022). In addition, the evaluation of decision quality showed that the proportion of the experimental group adopting the doctor-nurse–patient shared decision-making (SDM) model (70.0%) was much higher than that of the control group (30.0%), and it was also better than the control group in terms of decision-preference matching rate and knowledge compliance rate (all *p* < 0.05). In terms of decision-making efficiency, the decision confirmation time of the experimental group was also significantly shorter than that of the control group (*p* < 0.01) (Table 4).

**Table 4:** Vascular access distribution between the two groups [*n* (\%)].

Indicator	Control group (*n* = 60)	Experimental group (*n* = 60)	Statistic (χ2/t)	*p* value
Final type of vascular access				
AVF (Autologous Arteriovenous Fistula)	35 (58.3)	48 (80.0)	7.601	0.022
AVG (Arteriovenous Graft)	12 (20.0)	8 (13.3)		
CVC (Central Venous Catheter)	13 (21.7)	4 (6.7)		
Evaluation of decision-making role				
Patient-led	5 (8.3)	12 (20.0)	32.732	<0.001
Shared Decision-Making (SDM)	18 (30.0)	42 (70.0)		
Physician/Family-led	37 (61.7)	6 (10.0)		
Decision-Preference Match Rate	32 (53.3)	44 (73.3)	5.167	0.023
Decision Knowledge Achievement Rate	28 (46.7)	42 (70.0)	6.72	0.010
Decision Time (h)	38.5 ± 12.4	32.2 ± 8.6	−3.234	0.002

### 3.5 Comparison of the incidence of vascular access-related complications during 6-month follow-up

The results of 6-month follow-up after operation showed (see Table 4) that the incidence of multiple vascular access-related complications in the experimental group was significantly lower than that in the control group. Specifically, the experimental group exhibited a significantly lower incidence of vascular access stenosis (28.33% vs. 51.67%, *p* < 0.05), aneurysmal dilatation (31.67% vs. 56.67%, *p* < 0.05), and puncture-related circulatory failure (10.00% vs. 25.00%, *p* < 0.05) than the control group.

## 4 Discussion

This study constructed a nurse-led stepwise guided SDM model based on the Decisional Conflict Scale (DCS) and verified its application effect through a randomized controlled trial. The results showed that after intervention, the scores of the experimental group in all dimensions of DCS were significantly lower than those of the control group, indicating that this model can effectively alleviate patients’ decisional conflict and improve the scientificity and rationality of decision-making. This is mainly due to the precise positioning of the model for patients’ decision-making pain points: unlike the traditional “one-size-fits-all” health education model, this study first uses DCS to conduct a comprehensive assessment of patients’ decisional conflict, and then formulates personalized intervention strategies according to the scores of different dimensions (19). For patients with insufficient information, the nurse focuses on providing objective and easy-to-understand medical information through decision aids; for patients with unclear values, the nurse guides them to sort out their true needs through targeted questions, helping them balance the advantages and disadvantages of different vascular access options (20). This targeted intervention model not only reduces the psychological confusion of patients, but also enables patients to participate in the decision-making process more actively, which is consistent with the core concept of patient-centered medical care (21).

The significant improvement in the “information acquisition” and “value clarification” dimensions of the experimental group fully reflects the important role of decision aids in the SDM process. In clinical practice, many patients reject AVF not because of rational judgment, but because of excessive fear of “puncture pain” and “appearance changes,” as well as lack of understanding of the long-term complications of CVC (22). The “vascular access pros and cons comparison table” used in this study converts obscure medical parameters into vivid and easy-to-understand living indicators, which helps patients quickly grasp the characteristics of different vascular access types. At the same time, the nurse’s stepwise guidance enables patients to fully understand the long-term benefits of AVF and the potential risks of CVC (23), so that patients can make choices based on their own values and medical principles (24). This is also the main reason why the AVF selection rate of the experimental group is significantly higher than that of the control group: the patient’s choice is no longer passive acceptance, but an active choice based on full information, which not only improves the acceptance of AVF, but also lays a foundation for postoperative compliance (25, 26).

Compared with the control group, the experimental group had significantly higher decision satisfaction, knowledge compliance rate and proportion of doctor-nurse–patient shared decision-making, which strongly highlighted the crucial role of nurses in the SDM process. Nurses, as the most frequent medical staff in contact with HD patients, have a deeper understanding of the patients’ living habits, psychological state and value orientation, and can better establish a trusting relationship with patients (27). In this study, nurses act as “decision coaches” to bridge the information gap between doctors and patients: on the one hand, they translate professional medical knowledge into language that patients can understand, reducing the difficulty of patients’ information acquisition; on the other hand, they sort out the patients’ values and feedback them to doctors, so that the final decision can take into account both clinical feasibility and patient wishes, and achieve a high matching between decision and preference. Findings suggest that this two-way communication model can improve patient satisfaction with the decision-making process and facilitate collaborative patient-provider interactions, which may ultimately contribute to enhancing the overall quality of medical services (Table 5).

**Table 5:** Comparison of vascular access-related complications between the two groups within 6 months postoperatively [*n* (%)].

Type of complication	Control group (*n* = 60)	Experimental group (*n* = 60)	χ^2^/Fisher	*p* value
Catheter/Puncture site infection	9 (15)	4 (6.67)	—	0.239
Thrombosis/Lumen occlusion	17 (28.33)	10 (16.67)	2.342	0.126
Postoperative/Puncture site hematoma or bleeding	8 (13.33)	3 (5)	—	0.204
Access stenosis (Ultrasound monitoring)	31 (51.67)	17 (28.33)	6.806	0.009
Steal syndrome (Coldness/Pain in the hand)	2 (3.33)	0 (0.00)	—	0.496
Aneurysmal dilatation (Fistula)	34 (56.67)	19 (31.67)	7.603	0.006
Unplanned extubation/Non-standard discontinuation	1 (6.67)	0 (0.00)	—	1
Cannulation difficulty leading to circulatory failure	15 (25)	6 (10)	4.675	0.031

The significant decrease in preoperative anxiety (SAS) scores in the experimental group also has important clinical significance. Preoperative anxiety is a common psychological state of HD patients when facing vascular access establishment, which is mainly caused by the sense of loss of control over the unknown surgical results and the fear of complications (28). The nurse-led SDM model gives patients the right to participate in decision-making, making patients feel that they are the main body of treatment, thereby enhancing their sense of control (29). When patients clearly understand the reasons for choosing a certain vascular access and have psychological preparation for future puncture and maintenance, their stress level is likely to decrease, which is not only conducive to the smooth progress of the operation, but also helps to improve the patient’s postoperative psychological state and may facilitate a more favorable environment for early access maturation (5). Relevant studies have shown that reducing preoperative anxiety is often associated with better postoperative compliance, which further supports our observation of favorable clinical trends in the experimental group (30, 31).

The 6-month follow-up results showed that the experimental group had significant advantages in the control of vascular access stenosis, aneurysmal dilatation and puncture success rate, which highlights a potential downstream benefit of the nurse-led SDM model. It should be noted that the intervention of decisional conflict does not directly improve the surgical operation level, but may indirectly influence the patient’s postoperative prognosis by improving the patient’s “decision internalization” level and compliance (32). When patients highly recognize the vascular access they choose, they are likely to be more active in cooperating with early vascular exercise, protecting the puncture side limb, and identifying the early warning signals of complications. The lower puncture failure rate in the experimental group may be due to the detailed guidance on puncture cooperation in the decision aid process, which reduces vascular spasm caused by tension. While acknowledged as speculative, the reduction in the incidence of stenosis and aneurysm could partially reflect the patients’ more strict compliance with maintenance specifications and reduced non-standard compression. This hypothesizes that a high-quality decision-making process might offer an extended, behavioral “long-tail effect” by encouraging positive patient involvement—representing an important exploratory aspect of this study.

Due to the inherent inability to blind participants and intervention nurses to the educational allocation, performance bias represents a significant limitation in this study. The improved physical outcomes may partially reflect inadvertent differences in postoperative nursing intensity; nurses delivering the SDM intervention may have unconsciously provided closer monitoring or more rigorous puncture care during follow-up dialysis sessions. Furthermore, while we hypothesize that the intervention improved patient adherence to vascular access maintenance, adherence behaviors were not objectively quantified. Additionally, operating surgeon selection preferences and unmeasured baseline variations in patient vascular anatomy were not fully accounted for, and these factors independently influence fistula patency. Consequently, the improvements in complication rates must be interpreted with caution. Future rigorous trials controlling for specific surgical techniques, detailed anatomical parameters, and objective adherence metrics are warranted to isolate the direct physiological impact of SDM pathways.

Compared with previous similar studies, this study has obvious advantages. Murea et al. applied SDM in the selection of vascular access for HD patients, which improved the patient’s decision satisfaction to a certain extent (5), but failed to use a standardized scale to assess the patient’s decisional conflict, resulting in insufficient pertinence of the intervention (29, 33). Another study by Ostroff et al. developed a decision aid tool for vascular access selection (34), but did not involve the stepwise guidance of nurses, and the patient’s participation in decision-making was not high. In contrast, this study takes DCS as the core assessment tool, constructs a standardized nurse-led stepwise guided SDM model, and combines decision aids to form a closed-loop intervention of “assessment-intervention-decision-confirmation,” which makes the research design more scientific and the intervention measures more operable. At the same time, this study also evaluates the long-term effect of the model through 6-month follow-up, which makes the research results more reliable.

Despite the promising findings, this study has several limitations that warrant consideration. First, regarding the study design, the use of convenience sampling within a single center may introduce selection bias, potentially limiting the generalizability of the results to broader hemodialysis populations. Second, concerning the temporal scope, the 6-month follow-up period is insufficient to adequately assess the long-term impacts of the SDM model. Consequently, the long-term survival rate of the AVF, extended patient quality of life, and overall cost-effectiveness remain unexplored. Third, regarding mechanistic attribution, while we observed a reduction in medium-term postoperative complications (such as stenosis, aneurysmal dilatation, and circulatory failure), these clinical improvements cannot be unequivocally attributed to the SDM intervention alone. Our hypothesis that the SDM model reduces complications through enhanced “decision internalization” and subsequent positive behavior is plausible but indirect. Because this study lacked validated, objective metrics to monitor patient compliance with vascular access maintenance, the exact mechanistic link remains unverified.

Building on these limitations, future research should focus on conducting rigorous, multi-center randomized controlled trials with extended follow-up periods and the integration of objective adherence tracking. This will help isolate the direct physiological impact of SDM pathways and clarify the mechanism linking SDM to long-term access patency. Additionally, the delivery of the SDM model could be further optimized through digital health innovations—such as developing intelligent decision aids via WeChat mini-programs—to enhance accessibility, user convenience, and the overall efficacy of the intervention.

In conclusion, the nurse-led shared decision-making model constructed in this study demonstrates the capacity to identify patients’ decisional conflict, help alleviate preoperative anxiety, and improve short-term decision quality and satisfaction. By respecting patients’ values, this model supports the implementation of the “AVF first” principle and encourages postoperative maintenance compliance, while showing encouraging preliminary trends in early physical clinical outcomes. Overall, this approach can enhance the quality of nursing care and foster more collaborative patient-provider communication. While these preliminary findings are promising, further multi-center studies with extended follow-up periods are required to fully evaluate its impact on long-term patient prognosis and broader clinical applicability.

## Data Availability

The original contributions presented in the study are included in the article/Supplementary material, further inquiries can be directed to the corresponding author.

## Footnotes

1
http://www.chictr.org.cn
